# Reimagining Intermediate Care: Reflections From the Community Hospital of the Future (CHoF) Pilot in Singapore

**DOI:** 10.5334/ijic.9850

**Published:** 2025-11-20

**Authors:** Justin Guang Jie Lee, Qin Xiang Ng, Richard Wing Hong Chan, Jeffrey Jiang, Kelvin Wee Boon Koh

**Affiliations:** 1 Regional Health System Office, National University Health System, Singapore; 2 Saw Swee Hock School of Public Health, National University of Singapore and National University Health System, Singapore; 3 MOH Office for Healthcare Transformation, Singapore; 4 Jurong Community Hospital, Singapore

**Keywords:** intermediate care, integrated care, ageing population, community hospitals, system transformation

## Abstract

As Singapore’s population ages, community hospitals must evolve to meet increasingly complex care needs. In this perspective, we share reflections from leading the Community Hospital of the Future (CHoF) pilot at Jurong Community Hospital—a national initiative to enhance intermediate care. The pilot introduced proactive screening, expanded diagnostic capabilities, and intensified rehabilitation services. We reflect on the operational and policy challenges encountered, including fragmented data systems, workforce limitations, and financing gaps. The CHoF experience offers practical insights for other health systems seeking to strengthen sub-acute care as part of an integrated care strategy for ageing populations.

## Context and Aim

Singapore is experiencing one of the fastest rates of population ageing in Asia. By 2030, one in four Singaporeans will be aged 65 or older, putting immense pressure on a health system historically centred around acute hospitals [1]. Community hospitals, once viewed as convalescent or step-down facilities, are now being called upon to manage increasingly complex sub-acute and rehabilitative care needs [2]. In the United Kingdom (UK), intermediate care has helped lower hospital length of stay, prevented hospital admissions, and enhanced patient satisfaction [3]. Initiatives such as UK’s “Hospital at Home” and “Enhanced Rapid Response Service” have led the integration of acute and community services [4].

In Singapore, community hospitals provide sub-acute medical, nursing, and rehabilitative care for patients transitioning from acute hospital admission to home, with typical stays of two to four weeks [5]. However, despite their strategic importance, community hospitals in Singapore face challenges related to limited clinical capabilities, fragmented information systems, and workforce shortages. Most community hospitals work closely with acute hospitals, sharing electronic health records (albeit imperfectly) and coordinating discharge planning to ensure seamless transitions. Yet, the complex needs of older adults often exceed community hospitals’ current capacity, leading to prolonged stays and occasional re-admissions to acute hospitals [2]. Table 1 provides a snapshot of the strengths, weaknesses, opportunities, and threats (SWOT) facing Singapore’s community hospitals.

**Table 1:** SWOT analysis for community hospitals in Singapore.


STRENGTHS	WEAKNESSES

- Integrated care model with acute hospitals. - Close proximity to acute care for higher acuity for community hospitals that are co-located with acute hospitals. - Lower cost of care to healthcare system compared to acute hospitals. - Greater expertise in rehabilitation care and discharge planning amongst the multidisciplinary team.	- Wide variation in clinical capabilities across community hospitals, especially between co-located and standalone community hospitals without a neighbouring acute hospital partner. - Discharge delays due to social and logistical issues such as lack of rehabilitation potential or inadequate community support. - Disparity in access to advanced imaging across community hospitals - Reduced patient-clinical team ratio (including nurses and doctors) compared to acute hospitals. - Perceived unattractiveness for healthcare care professionals to work in community hospitals compared to acute hospitals. - Limited care capabilities in community hospitals necessitate the transfer of patients with deteriorating clinical conditions back to acute hospitals for advanced medical management. - Fragmented electronic health records (EHR) between acute and community hospitals slow down patient transfers and decision-making.

**OPPORTUNITIES**	**THREATS**

- Expansion of clinical capabilities to manage emerging clinical issues to minimise re-admissions to acute hospitals. - Subsidy expansion and improved access for all appropriate advanced imaging. - Direct admissions from community. - Increased collaboration with community partners. - Leveraging machine learning for early patient identification and propensity scoring to streamline admissions, optimise bed use, and improve care transitions. - Research on reducing re-admissions and improving outcomes.	- Expansion of clinical capabilities in community hospitals depends on securing adequate staffing resources to manage the anticipated increase in clinical workload. - Process inefficiencies remain as potential roadblocks to expanded care models, particularly the complex procedures for patient transfers and the complicated workflows requires to access acute hospitals’ imaging and laboratory services. - Greater demand and more complex care as population ages. - Current funding models are insufficient to support the expanding scope and enhanced capabilities expected of community hospitals.


To address these gaps, in 2024, the Ministry of Health launched the Community Hospital of the Future (CHoF) pilot at Jurong Community Hospital (JCH) to explore how intermediate care could be reimagined to meet these demands [6]. As members of the design, implementation, and evaluation team, we offer reflections on the pilot’s innovations, its challenges, and the broader implications for integrated care.

## Description of the Innovation

**Figure 1:**
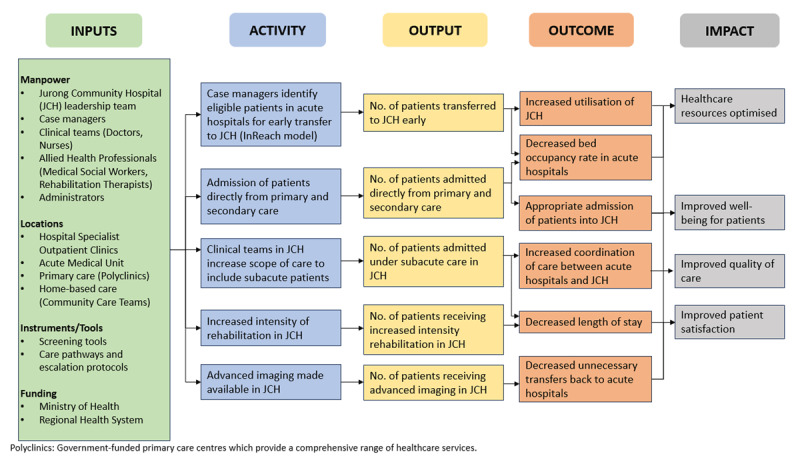
CHoF’s adapted programme theory to integrate care for the population.

As illustrated in Figure 1, the CHoF pilot introduced four key thrusts aimed at strengthening the role of community hospitals. Referencing international intermediate care models from UK, Ireland and New Zealand, the programme theory emphasises close collaboration with Ng Teng Fong General Hospital (NTFGH) clinical teams to optimise patient status and coordinated care management throughout the transfer process.

Firstly, the InReach model emphasises proactive identification and early screening of patients suitable for community hospital care. Historically, patient referrals to JCH were passive, often occurring late in an acute admission. Under the pilot’s InReach model, specially trained senior nurses (case managers) actively screen potential candidates in NTFGH for early transfer to JCH. This innovative approach aims to streamline the transition, shortening overall length of stay while preserving continuity of care. The programme emphasises close collaboration with NTFGH clinical teams to optimise patient status and coordinated care management throughout the transfer process.

Secondly, expanded points of entry, where beyond receiving patients from acute hospitals (including emergency department), JCH can now directly admit suitable individuals from primary care, specialist outpatient clinics, or community care teams. By broadening admission sources, the pilot hopes to make the process more seamless and intercept health issues before they escalate, decreasing avoidable admissions to acute hospitals.

Thirdly, expanded scope of practice, where JCH is now admitting patients without a definite diagnosis hence piloting advanced imaging (e.g., computed tomography, magnetic resonance imaging) and subacute services. Previously, community hospital patients requiring these investigations had to be transferred back to acute hospitals, causing inefficiencies and additional risks. Delivering these services on-site potentially reduces delays, alleviates patient discomfort, and enhances the clinical depth of community care.

Fourthly, intensified rehabilitation where the CHoF pilot expands physiotherapy and occupational therapy services, exploring state-of-the-art rehabilitation robots to improve patient outcomes.

These changes seek to relieve pressure on acute hospitals, which face significant bottlenecks, and ensure that patients receive timely, cost-effective, and person-centred care. The pilot is being rolled out in phases, with interim evaluations at six and twelve months. A modified programme theory underpins the initiative (see Figure 1). Process indicators include patient safety metrics, recruitment rates, and average length of stay; while outcome indicators include readmissions to acute settings within 72 hours and overall patient satisfaction. Data collection is still ongoing at the time of writing.

## Our Role and Perspective

We were involved in the design and implementation of the CHoF pilot as clinicians, public health practitioners, and policy advisors. Our work spanned programme conceptualisation, development of clinical protocols, outcome monitoring, and policy translation. This positioning enabled us to observe first-hand the structural, operational, and cultural changes needed to advance integrated intermediate care.

## Discussion and Reflection

1. **Integration Is More Than Co-location:** While JCH is co-located with a tertiary acute hospital (NTFGH), we found that integration does not occur automatically. Different care teams, information systems, and organisational cultures created friction in care transitions. Achieving integration requires shared clinical protocols, interoperable systems, and mutual accountability across settings.
  Integration is complete only when the patient’s home is part of the care continuum, with community hospitals forming the platform for onward transition. Existing discharge planning process incorporates multidisciplinary collaboration, community care coordination, and linkage with primary care and social services. National initiatives, including the National Transitional Care Programme and Community Health Posts, help maintain functional improvements and prevent re-admissions, similar to Ireland’s community-centred Early Supported Discharge model where care is delivered in homes or community clinics [7].
2. **Workforce Remains a Major Bottleneck:** Expanding the scope of community hospital services heightened demand for staff with experience in managing sub-acute and complex cases. However, community hospitals in Singapore have historically struggled to attract and retain talent. Perceived as less prestigious than acute hospitals, community hospitals offer fewer career advancement opportunities and often have lower staffing ratios. Addressing these workforce challenges requires a multipronged policy approach such as introducing financial incentives and career pathways to encourage rotations or permanent placements in community hospitals; promoting interdisciplinary training programs to prepare healthcare workers for the expanded scope of practice required in sub-acute care settings; and developing technology-enabled solutions, such as AI-driven patient screening tools, to reduce administrative burdens and improve workforce productivity.
3. **Financing Models Must Evolve:** The enhanced services under CHoF such as advanced imaging, longer stays, and intensive rehabilitation imply higher costs. Current Diagnosis-Related Group (DRG) models may under-reimburse community hospitals for the care they now provide. Funding models need to shift toward bundled payments and value-based incentives that reward functional recovery and reduced readmissions. Policymakers must assess whether these investments in community hospitals will result in net cost savings across the healthcare system by reducing reliance on acute hospitals and long-term residential care. Potential financing strategies include developing bundled payment models that incentivise integrated care pathways across acute and community settings; introducing value-based payment schemes that reward improved functional outcomes and reduced readmissions; and expanding subsidies for community hospital services to ensure affordability for patients and families.
4. **Social Determinants of Health Cannot Be Ignored:** Anecdotally, delayed discharges were often due to non-clinical factors, e.g., lack of caregiver support, housing instability, or delayed deployment of home-based services, similar to findings elsewhere [8]. This reinforced our view that intermediate care must be embedded within broader social support systems. Integrated care cannot succeed without investments in social partnerships and community-based services.
5. **Normative Integration and Cultural Change:** Building an effective integrated care ecosystem requires cultivating shared values, mutual trust, and collective ownership across acute, community, and primary care sectors. Recognising sub-acute care as equally important as acute care is vital for sustainability. CHoF findings emphasise the importance of cultural transformation, achievable through collaborative training, cross-posting of staff, and shared performance metrics.

## Conclusion and Recommendations

Singapore’s CHoF pilot represents a bold step toward reimagining intermediate care for an ageing population. By proactively identifying patients for early transfer, expanding clinical capabilities, and intensifying rehabilitation services, the pilot seeks to alleviate acute hospital pressures and improve patient outcomes. Yet, the pilot also surfaces critical policy questions that must be addressed to ensure its long-term viability. Workforce development, sustainable financing, and robust system integration are essential pillars of success. There are no easy answers and addressing these questions would require a balanced, systems-level approach that recognises the multifaceted care needs and determinants of health in older populations, from biological to social and environmental factors. As health systems worldwide confront similar demographic and fiscal pressures, the lessons from Singapore’s CHoF initiative offer valuable insights into how intermediate care models can evolve to meet the complex needs of ageing societies. Future research should focus on rigorous evaluations of the CHoF pilot’s cost-effectiveness, patient outcomes, and workforce implications. Only through such evidence can policymakers make informed decisions on scaling and sustaining this transformative model of care.
